# A GPU-Based Gibbs Sampler for a Unidimensional IRT Model

**DOI:** 10.1155/2014/368149

**Published:** 2014-10-30

**Authors:** Yanyan Sheng, William S. Welling, Michelle M. Zhu

**Affiliations:** ^1^Educational Measurement and Statistics, Department of Educational Psychology & Special Education, Southern Illinois University, Carbondale, IL 62901, USA; ^2^Department of Computer Science, Southern Illinois University, Carbondale, IL 62901, USA

## Abstract

Item response theory (IRT) is a popular approach used for addressing large-scale statistical problems in psychometrics as well as in other fields. The fully Bayesian approach for estimating IRT models is usually memory and computationally expensive due to the large number of iterations. This limits the use of the procedure in many applications. In an effort to overcome such restraint, previous studies focused on utilizing the message passing interface (MPI) in a distributed memory-based Linux cluster to achieve certain speedups. However, given the high data dependencies in a single Markov chain for IRT models, the communication overhead rapidly grows as the number of cluster nodes increases. This makes it difficult to further improve the performance under such a parallel framework. This study aims to tackle the problem using massive core-based graphic processing units (GPU), which is practical, cost-effective, and convenient in actual applications. The performance comparisons among serial CPU, MPI, and compute unified device architecture (CUDA) programs demonstrate that the CUDA GPU approach has many advantages over the CPU-based approach and therefore is preferred.

## 1. Introduction

Item response theory (IRT) is a popular approach used for describing probabilistic relationships between correct responses on a set of test items and continuous latent traits (see [1–4]). In addition to educational and psychological measurement, IRT models have been used in other areas of applied mathematics and statistical research, such as US Supreme Court decision-making processes [5], alcohol disorder analysis [6–9], nicotine dependency [10–12], multiple-recapture population estimation [13], and psychiatric epidemiology [14–16], to name a few.

In IRT, the influence of items and persons on the responses is modeled by distinct sets of parameters so that the probability of a correct response to an item is a function of the person's latent trait, *θ*, and the item's characteristics, **ξ**; that is,

(1) $$\begin{array}{c} P\left(y=\text{correct}\right)=f\left(\theta ,\xi \right). \end{array}$$

In its original form, *θ* is used to denote person's unobserved “ability.” Certainly, for psychological scales and other applications, it might be better labeled as “mood,” “attitude,” “depression,” and so forth, depending on what the instrument is intended to measure. The model assumes one *θ* parameter for each person and is commonly referred to as the unidimensional model, signifying that each test item measures some facet of the unified latent trait. In this sense, IRT models are usually related to factor analysis models despite their many differences [17].

The mathematical form of the model has made IRT a more powerful psychometric theory than the traditional classical test theory (which uses sum scores to estimate latent person traits), and its advantages have been well documented in [18–20]. Specifically, IRT models offer the ability to “(a) rigorously study how items function differently across examinee populations, (b) place individuals who have responded to different items onto a common scale, (c) derive individual scores that have good psychometric properties, (d) more thoroughly understand the psychometric properties of items and scales through inspection of item parameters and information functions, (e) create order in various research fields by having a common item pool and latent scale for key constructs, and (f) develop computerized adaptive testing (CAT) systems or static short-forms for precise and efficient assessment of individual differences” [21, page 212].

Although recent advances in computer technology and development of estimation algorithms have made the application of IRT accessible to a wider range of users, problems exist in the efficiency of current estimation tasks with IRT models. We describe them in the following section.

### 1.1. Gibbs Sampling for IRT Models

Given that IRT is modeled by distinct sets of parameters, a primary concern associated with IRT research has been on parameter estimation, which offers the basis for the theoretical advantages of IRT. Specifically, of concern are the statistical complexities that can often arise when item and person parameters are simultaneously estimated (see [1, 22–24]). More recent attention has focused on the fully Bayesian estimation where Markov chain Monte Carlo (MCMC, [25, 26]) simulation techniques are used. Albert [27] applied Gibbs sampling [28], one of the most efficient MCMC algorithms, to a unidimensional IRT model, namely, the two-parameter normal ogive (2PNO; [29]) model.

The fully Bayesian approach for estimating IRT models is both memory and computationally expensive, which further limits its actual applications. Typically, item response data are based on *n* subjects' responses to *k* items at one occasion, and a Markov chain requires 5,000 to 10,000 iterations to reach convergence for such IRT models. Each implementation of the algorithm would take five or more minutes to complete the computation by a single desktop when *n* and *k* are not sufficiently large (e.g., *n* = 1000, *k* = 10) [30]. This fact makes it impractical for users to utilize the algorithm for various applications of IRT. For example, in standardized testing situations, each instrument can consist of 100 or more items, and the number of participants during each administration can go beyond 10,000. The implementation of Gibbs sampling would take considerably long time. This is not practical with testing companies, as their primary consideration is to promptly report accurate trait estimates to the participants considering the frequency of handling the estimation of multiple tests/datasets. Another example is with the test development or scale construction where item analysis is a necessary step before individual items are included in an instrument. The common practice is to collect data, estimate item parameters, analyze item performances, and modify the instrument accordingly. This procedure repeats until all items have desirable psychometric properties. The efficiency of the algorithm is hence important in delivering prompt item estimates, without which item analysis is not possible. Other examples include using IRT (1) to diagnose patients for certain mental disabilities in psychiatry where the urgency of starting treatment of a concerning disability is essential, (2) to calibrate item parameters for a CAT system where a large item pool with sufficient numbers of good quality items is required, and (3) in the massive open online courses (MOOCs) where sample sizes and test frequencies are much larger.

In addition to these applications, the computation expense limits researchers in conducting Monte Carlo studies where a large number of replications are desirable. In the IRT literature, simulation studies commonly utilize 25 replications only [31], which makes it difficult to empirically evaluate the property of the population distribution of the model parameter. Even with such a small number of replications, the entire execution takes weeks or even months to finish. The delayed research findings would in turn prevent the advance of IRT research in developing more complicated IRT models. In general, the serial implementation of the Gibbs sampler is limited in both practical applications and theoretical developments. Consequently, achieving a considerable speedup and making less requirement on the memory size with well-designed parallel algorithms on an inexpensive and convenient execution platform would make it more practical for researchers or practitioners to implement such an IRT model using MCMC.

### 1.2. High Performance Computing

High performance computing (HPC) employs supercomputers, computer clusters, and graphics processors to tackle problems with computing and memory intensive computations. HPC utilizes the concept of parallel processing to run programs in parallel and achieve a much smaller execution time with high efficiency and low overhead.

(*1) MPI Standard.* Many large-scale applications run on HPC machines through the message passing interface (MPI) standard to achieve a better performance. Previous studies have applied MPI to implement Gibbs sampling for the 2PNO IRT model [32, 33]. However, parallel computing is known to excel at tasks that rely on the processing of discrete units of data that are not heavily interdependent. Given the high data dependencies in a single Markov chain for IRT models, such as the dependency of one state of the chain to the previous state and the dependencies among the data within the same state, the implementation of MPI for this problem requires domain decomposition [34] of data matrices while minimizing the communication overhead among processors. Pastias et al. [32, 33] have demonstrated the advantage of parallel computing over the serial implementation, but with MPI, a speedup of only up to 5× or 6× could be achieved in their experimented conditions with efficiency gradually dropping as more processors are added due to the rapid growth in the communication overhead.

(*2) Massive Core GPU Computing.* CUDA-enabled graphic processing units (GPU) is gaining a growing research interest for data decomposition-based parallel applications. As of 2012, the peak floating-point throughput of many-thread GPU is 10 times that of a multicore CPU. Such a big gap between CPU and GPU is due to two factors. First, the design of CPU is optimized for sequential algorithms with a complicated control logic and a large cache. Latency can be reduced by such designs but the throughput will be sacrificed. Second, the memory bandwidth of delivering data from the memory to the processor is about six times faster for GPU than that of CPU, for which the bandwidth usually serves as the bottleneck in many applications [35]. Hence, even a single GPU card is capable of delivering much improved performances.

The data size and the data-parallelism nature of the MCMC procedure with a high throughput requirement make GPU an ideal platform for a fast and efficient execution. A typical GPU program utilizes thousands of threads simultaneously and can achieve an extremely high system throughput. On the contrary, a high-end multicore microprocessor CPU typically has only four to eight cores and multiple megabytes of on-chip cache for strong sequential code performance.

In view of the above, the CUDA-enabled GPU can potentially accelerate the speed for implementing MCMC with an IRT model, and, as the data size increases, the benefit of using GPU would increase. To the best of our knowledge, generic software for implementing MCMC techniques such as BUGS [36] or JAGS [37] does not currently provide CUDA support. Although R [38] has many parallel computing packages, the only package that implements CUDA with Bayesian modeling, cudaBayesreg [39], is specifically limited to fMRI data analysis. Hence, the purpose of this study is to develop a GPU-based high performance Gibbs sampling algorithm for the 2PNO IRT model and further compare it with the CPU-based program.

The remainder of the paper is organized as follows.  Section 2 illustrates the approach we propose in the present study to implement the MCMC algorithm using CUDA. In Section 3, the performance of the proposed parallel algorithm is investigated by comparing it with the serial CPU algorithm and further with the parallel algorithm using MPI developed in [33]. A real data example is provided in Section 4 to illustrate the program developed for implementing the proposed parallel algorithm. Finally, a few remarks are made in Section 5.

## 2. Methodology

This study was performed using a Tesla K20c GPU on an Intel Core 2 Quad CPU with 8 GB of RAM. For the purpose of comparisons, the CPU-based MPI algorithm developed by Sheng and Rahimi [33] was carried out using the Maxwell Linux cluster, a cluster with 106 processing nodes. Maxwell uses the message-passing model via the MPICH framework implementation. Each node on the cluster has an Intel Xeon dual CPU quad-core processor clocked at 2.3 GHz, 8 GB of RAM, 90 TB storage, and a Linux 64 bit operating system.

### 2.1. Serial Algorithm

The 2PNO IRT model provides a fundamental framework in modeling the person-item interaction by assuming one latent dimension. Let **y** = [*y*_*ij*_] denote a matrix of *n* responses to *k* items where *y*_*ij*_ = 1 (*y*_*ij*_ = 0) if the *i*th person answers the *j*th item correctly (incorrectly) for *i* = 1,…, *n* and *j* = 1,…, *k*. The probability of person *i* obtaining a correct response to item *j* is then defined for the 2PNO model as

(2) $$\begin{array}{c} P\left(y_{ij}=1\right)=\Phi \left(\alpha_{j}\theta_{i}-\beta_{j}\right)=\overset{\alpha_{j}\theta_{i}-\beta_{j}}{\underset{-\infty }{\int }}\frac{1}{\sqrt{2\pi }}e^{-t^{2}/2}dt, \end{array}$$

where *α*_*j*_ and *β*_*j*_ denote item slope and intercept parameters and *θ*_*i*_ denotes the continuous person trait parameter.

The Gibbs sampler involves updating three sets of parameters in each iteration, namely, an augmented continuous variable *Z*_*ij*_ (which is positive if *y*_*ij*_ = 1 and negative if *y*_*ij*_ = 0), the person parameter *θ*_*i*_, and the item parameters **ξ**_*j*_, where **ξ**_*j*_ = (*α*_*j*_, *β*_*j*_)′ from their respective full conditional distributions; namely,

(3) $$\begin{array}{c} Z_{ij}\;|\;\cdot \sim \left\{\begin{array}{cc} N_{\left(0,\infty \right)}\left(\alpha_{j}\theta_{i}-\beta_{j},1\right), & \text{if}\;\;y_{ij}=1\\ N_{\left(-\infty ,0\right)}\left(\alpha_{j}\theta_{i}-\beta_{j},1\right), & \text{if}\;\;y_{ij}=0, \end{array}\right. \end{array}$$

(4) $$\begin{array}{c} \theta_{i}\;|\;\cdot \sim N\left(\frac{\sum_{j}\left(Z_{ij}+\beta_{j}\right)\alpha_{j}}{\sum_{j}\alpha_{j}^{2}},\frac{1}{\sum_{j}\alpha_{j}^{2}}\right), \end{array}$$

(5) $$\begin{array}{c} \xi_{j}\;|\;\cdot \sim N\left({\left(x^{\prime }x\right)}^{-1}x^{\prime }Z_{j},{\left(x^{\prime }x\right)}^{-1}\right)I\left(\alpha_{j}>0\right), \end{array}$$

where **x** = [***θ***, −1], assuming *θ*_*i*_ ~ *N*(0,1), *α*_*j*_ > 0 and *p*(*β*_*j*_) ∝ 1 (see, e.g., [27, 30]).

Hence, with starting values ***θ*** ^(0)^ and **ξ** ^(0)^, observations (**Z** ^(*ℓ*)^, ***θ*** ^(*ℓ*)^, **ξ** ^(*ℓ*)^) can be simulated from the Gibbs sampler by iteratively drawing from their respective full conditional distributions as specified in (3), (4), and (5). To go from (**Z** ^(*ℓ* − 1)^, ***θ*** ^(*ℓ* − 1)^, **ξ** ^(*ℓ* − 1)^) to (**Z** ^(*ℓ*)^, ***θ*** ^(*ℓ*)^, **ξ** ^(*ℓ*)^), it takes three transition steps as follows.

(1) Draw **Z** ^(*ℓ*)^ ~ *p*(**Z**∣***θ*** ^(*ℓ* − 1)^, **ξ** ^(*ℓ* − 1)^).
(2) Draw ***θ*** ^(*ℓ*)^ ~ *p*(*θ*∣**Z** ^(*ℓ*)^, **ξ** ^(*ℓ* − 1)^).
(3) Draw **ξ** ^(*ℓ*)^ ~ *p*(**ξ**∣**Z** ^(*ℓ*)^, ***θ*** ^(*ℓ*)^).

This iterative procedure produces a sequence of (***θ*** ^(*ℓ*)^, **ξ** ^(*ℓ*)^), *ℓ* = 0,…, *L*. To reduce the effect of the starting values, early iterations in the Markov chain are set as burn-ins to be discarded. Samples from the remaining iterations are then used to summarize the posterior density of item parameters **ξ** and ability parameters ***θ***.

### 2.2. GPU Implementation and Optimization

**Figure 1:**
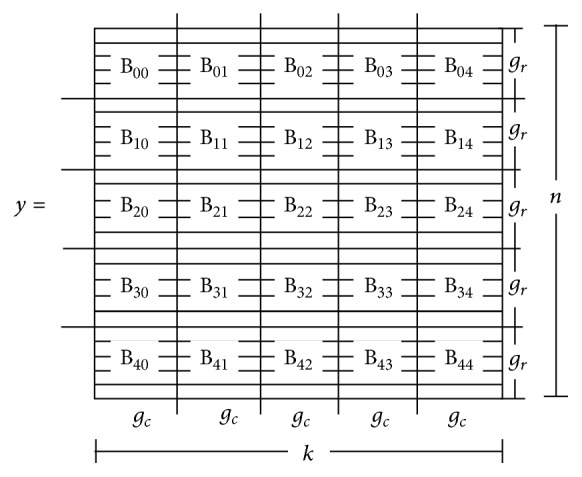
Decomposition of the data matrix **y** over a grid of (*r* = 5)×(*c* = 5) blocks.

**Figure 2:**
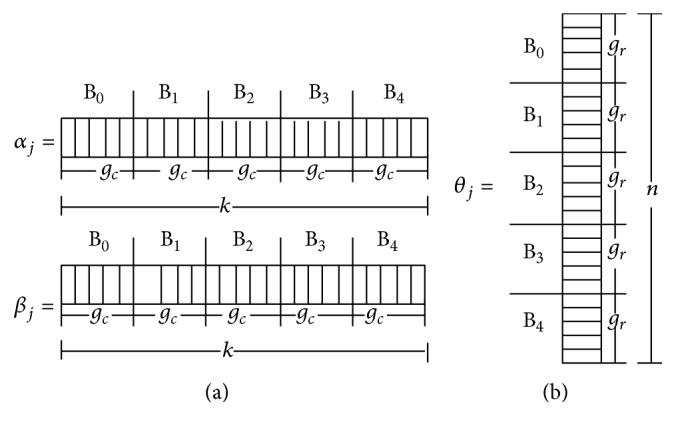
Decomposition of item parameters (a) and person parameters (b) over a list of *r* = 5 or *c* = 5 blocks.

The GPU-based parallel algorithm begins with copying the data matrix **y** to the device, which then assumes the tasks of updating model parameters *θ*_*i*_, *α*_*j*_, and *β*_*j*_ and calculating results. Using the triple chevron notation, we defined a kernel per update to specify the number of blocks and the number of threads per block for decompositions of the data matrix and model parameters. Hence, each kernel has a random state indexed in a grid or a list. Specifically, the data matrix **y**, which is of size *n* × *k*, was decomposed over a two-dimensional grid of *r* × *c* blocks with a defined number of threads (see Figure 1). This way, each block on the device receives a submatrix **y**_*B*_*ij*__ of size *g*_*r*_ × *g*_*c*_, where *g*_*r*_ = *n*/*r* and *g*_*c*_ = *k*/*c*. In addition, each item (person) parameter was decomposed over a list of *r* (*c*) blocks as depicted in Figure 2.

The algorithm was implemented in ANSI C with utilization of the cuRAND library [40] for random number generations and normal cumulative densities. Specifically, we employed the curand_normal2_double device API method, which uses the Box-Muller transform to generate two pseudorandom numbers at once. This is more efficient than generating a single value with each call. In addition, using vector types improves the efficiency of memory access because fewer accesses would be needed for the same amount of data handled.

For more detailed implementation, see Figure 11 where a basic control diagram is provided between CPU host and GPU device for updating various variables in the algorithm. Specifically, after the initial matrices (e.g., dev_Z), vectors (e.g., dev_AVZ, dev_GVZ), and input values (dev_Y) are stored in the device memory with random states allocated (rngStatesA, rngStatesB, and rngStatesC), the Gibbs sampler begins. The first update is in the kernel of updating **Z** (calcZ), which decomposes the data matrix **y** on a two-dimensional grid and calculates the augmented data **Z** (see Figure 1). This kernel requires passing the pointer to the random state matrix on the device (rngStatesA). Calculating the mean for *θ* (calcMN) is a separate kernel that is decomposed on a one-dimensional list of blocks with the size of *r* (see Figure 2). Updating *θ* (calcTH) is decomposed similarly but requires passing a pointer to a vector of random states on the device (rngStatesB). Updating *α* and *β* (calcAG) is decomposed on a one-dimensional list of blocks with the size of *c* (see Figure 2). This update also requires a random state for each block of the decomposed items (rngStatesC). Calculating the posterior estimates for item or person parameters (calcIS, calcPS), performed at the end of all the iterations, is also parallelized using a one-dimensional list of *c* or *r*. The program stops when the device sends all the results back to the host.

It is noted that the update of *θ* has been optimized using the Thrust library [41], which provides templates for various parallel linear algebra algorithms with improved efficiency. With the use of two custom defined C structure operators (one for vector addition and the other for inverse vector multiplication), a transform-reduce method from the Thrust library was successfully implemented to improve the performance when operating **x**′**x**, a 2 × *n* by *n* × 2 matrix multiplication, in (5).

In this study where data sizes have been determined in all the experimented conditions as described in Section 3, statically allocating at compile time was adopted due to its simplicity and efficiency in memory addressing of two-dimensional arrays and optimal memory alignment [42]. The compiler catches the exception when available memory is exceeded. When decomposing an MCMC algorithm using CUDA, each running kernel requires its own random state to grab the next random number in its sequence within a single block of each update. Therefore, after initial values are sent from the host to the device, a kernel must be executed to allocate random states in a vector or matrix for each similar kernel update. Adequate error checking is also performed on each call of the CUDA kernel.

### 2.3. Performance Analyses

In order to investigate the benefits of the proposed GPU-based parallel solution against its serial and MPI counterparts, experiments were carried out in which tests with *n* persons (*n* = 500,1000,2000,5000,10000) and *k* items (*k* = 20,50,100,200) were considered. In each experiment, Gibbs sampling was implemented to run a single Markov chain with a total of 10,000 iterations using (1) serial algorithm, (2) MPI with 10 processing nodes, (3) MPI with 20 processing nodes, and (4) CUDA with a single GPU card. They were evaluated using the execution time as well as the relative speedup, which is defined as

(6) $$\begin{array}{c} S=\frac{T_{S}}{T_{P}}, \end{array}$$

where *T*_*S*_ is the execution time for the fastest sequential algorithm and *T*_*P*_ is that for the parallel algorithm.

## 3. Results

**Figure 3:**
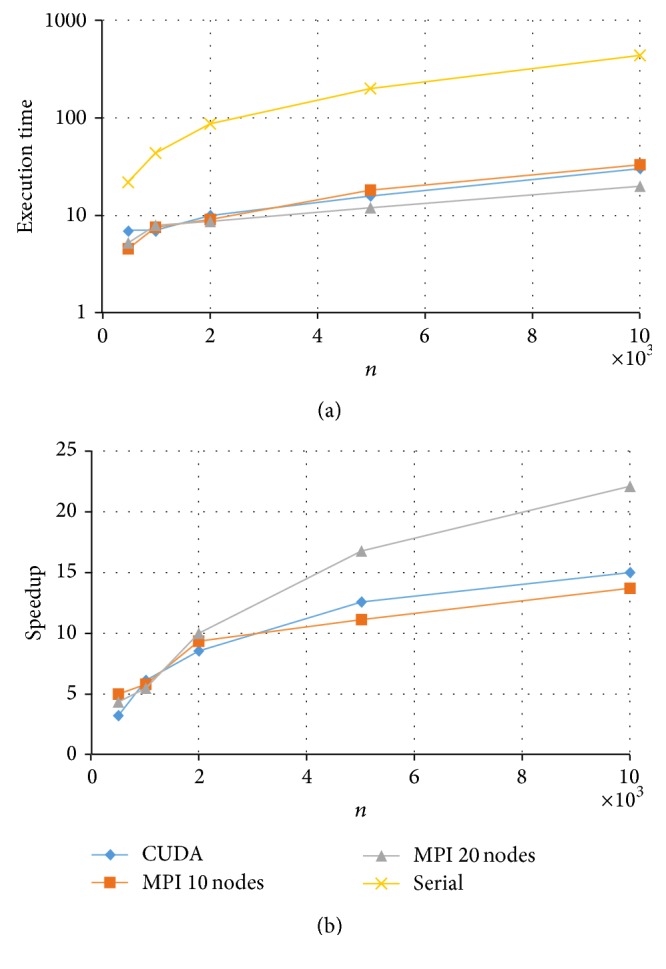
Execution time and speedup for implementing MPI and CUDA parallel programs of Gibbs sampling for tests with *k* = 20 items.

**Figure 4:**
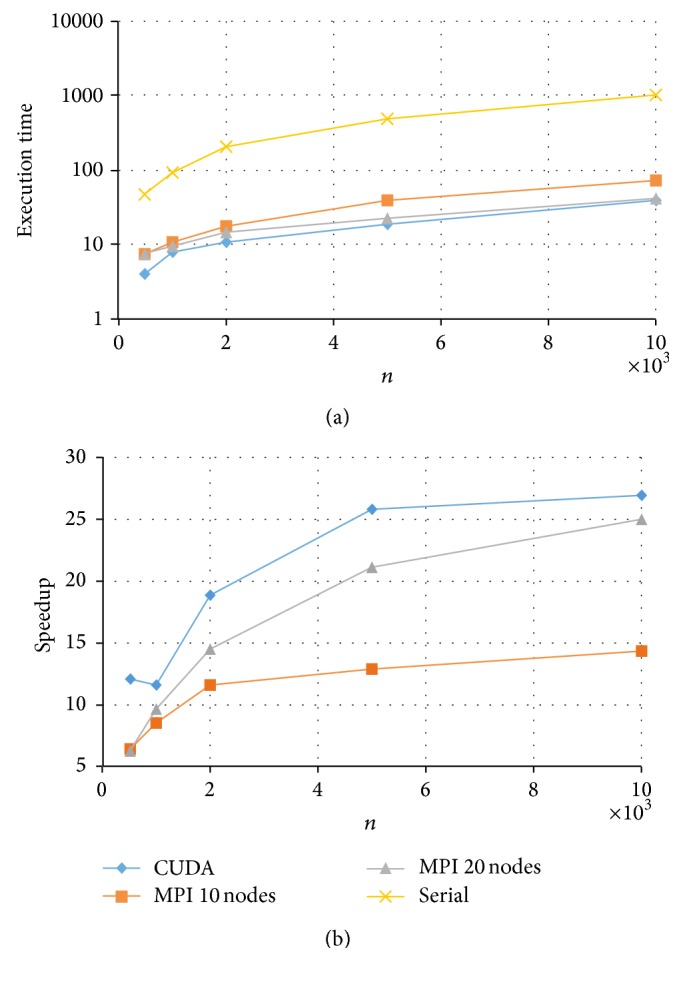
Execution time and speedup for implementing MPI and CUDA parallel programs of Gibbs sampling for tests with *k* = 50 items.

**Figure 5:**
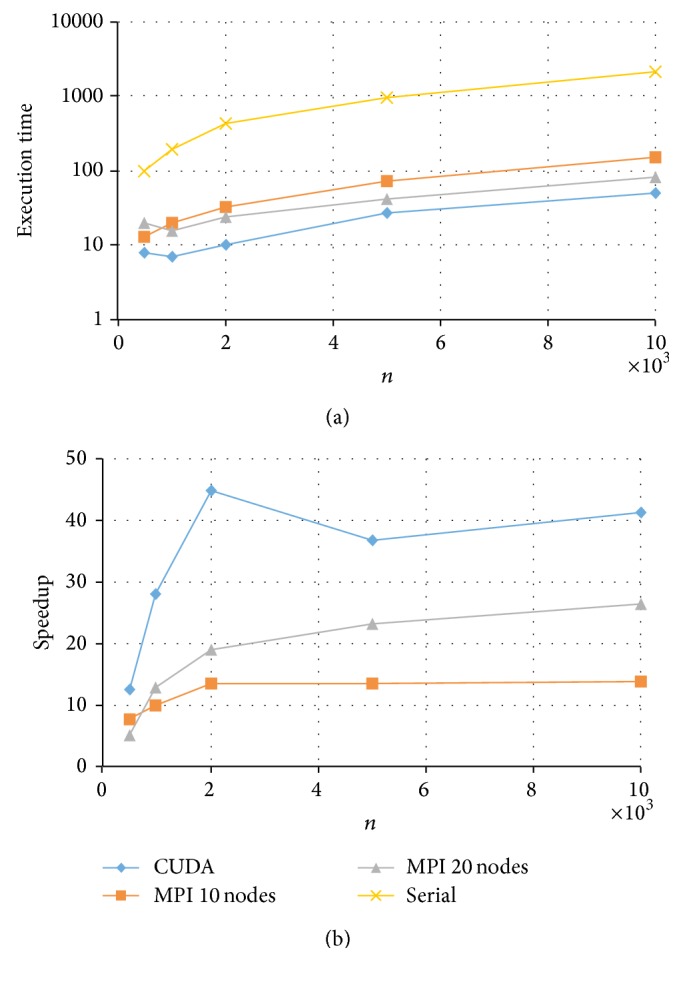
Execution time and speedup for implementing MPI and CUDA parallel programs of Gibbs sampling for tests with *k* = 100 items.

**Figure 6:**
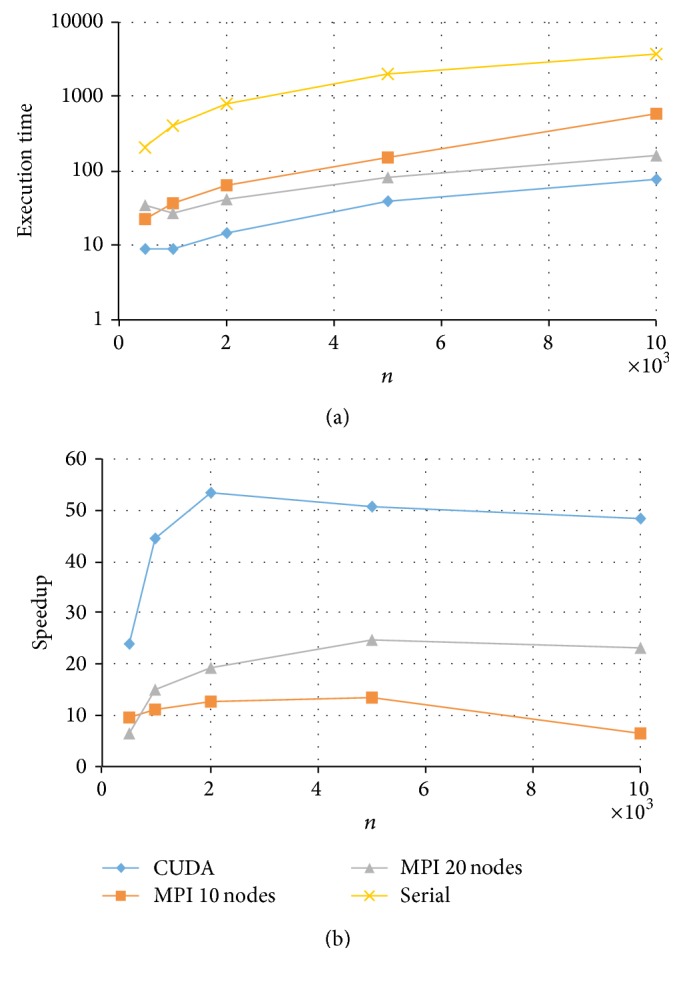
Execution time and speedup for implementing MPI and CUDA parallel programs of Gibbs sampling for tests with *k* = 200 items.

Results for the fully crossed 5 × 4 = 20 experiments are summarized in Figures 3 to 6. Note that the CPU-based values represent the average of ten replications. As expected, parallel programs had a shorter execution time than the serial program in all the experimented conditions. Under these conditions, the GPU-based program could achieve a speedup of up to 50× while the MPI program achieved a speedup of up to 20×. For example, for the data size of *k* = 200 and *n* = 5000, the serial implementation took more than half an hour, the MPI with 20 nodes took about 81 seconds, and the CUDA implementation took only 40 seconds to complete a Markov chain (see Figure 6).

**Figure 7:**
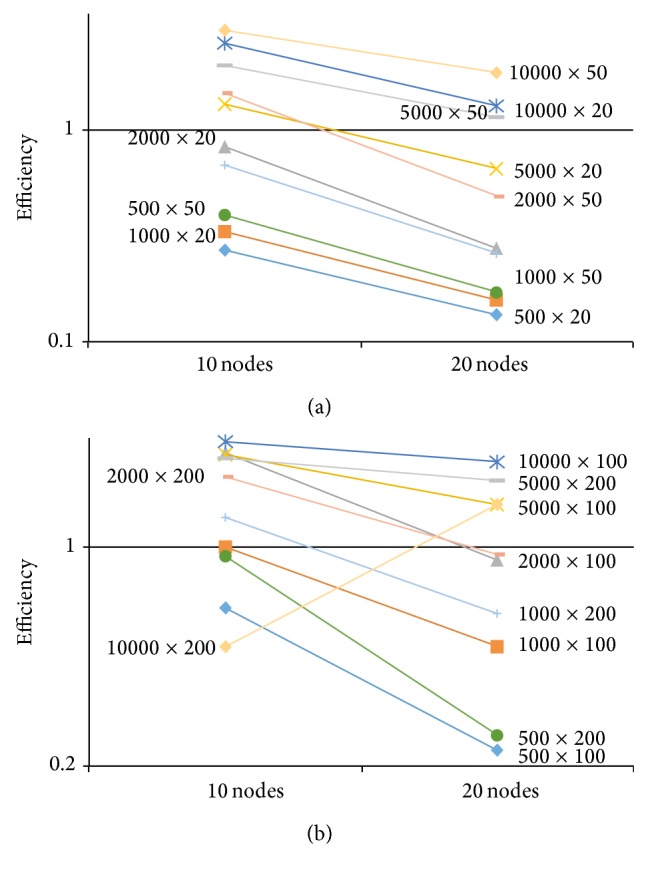
Efficiency for implementing MPI algorithms with 10 and 20 nodes for data sizes of *n* × *k*.

Comparing the two HPC platforms, MPI versus CUDA, we observe a clear pattern that with a test length of *k* = 20, both MPI and CUDA resulted in similar execution times with a slight advantage to MPI (with 20 nodes) especially for data with a larger *n* (see Figure 3). On the other hand, when test length *k* increased, CUDA showed a much shorter computing time and thus a higher speedup (see Figures 4 through 6). The reason is due to the fact that the MPI algorithm developed by Sheng and Rahimi [33] uses a row-wise decomposition and consequently the communication size depends on the test length *k*. It follows that when *k* is large (e.g., *k* > 20), the communication overhead overshadows the computation speedup. This can be further demonstrated by assessing the relative efficiency of the MPI program in managing the computational resources, which is defined as

(7) $$\begin{array}{c} E=\frac{T_{S}}{PT_{P}}, \end{array}$$

where *P* is the number of available processing nodes, and *T*_*S*_ and *T*_*P*_ are as defined in (5). The efficiency values using MPI with 10 and 20 processing nodes are plotted in Figure 7 for all the *n* × *k* experimented conditions. Given the reasons stated previously, we observe the general pattern that, for the same data size, increasing the number of processing nodes from 10 to 20 generally decreases the relative efficiency. Hence, the fact that the overhead of the MPI algorithm such as data communication cost grows faster than the computation speedup leads to the result that increasing the number of the MPI nodes reduces efficiency. The amount of decrease reduces when the data size gets large, especially for tests with *k* > 20. In addition, with a fixed number of nodes, the efficiency improves with the increase of *n* or *k* because the computational gain overweighs the overhead loss. Given the nature of data decomposition in the MPI algorithm, an increased *n* tends to have a larger effect on the relative efficiency than an increased *k*. It is also observed that the efficiency exceeds 1 for larger data sizes, which is due to the fact that, when there is insufficient memory for the problem to execute on a sequential program, the memory on multiple processing nodes can be utilized to overcome this limit. This further illustrates that parallel algorithms can utilize a faster memory for a better performance.

Consequently, MPI is not a scalable approach when *n* and/or *k* increases. On the contrary, the developed CUDA program demonstrates a much improved scalability and efficiency when the data size goes up and further allows us to investigate even larger data sizes with high accuracy.

With respect to the CUDA implementations, for *k* = 20 or *k* = 50, the relative speedup kept increasing for increased sample sizes *n* (see Figures 3 and 4). This suggests that the developed CUDA program can scale up in both *k* and *n*, which makes it possible to fit the 2PNO IRT model to large-scale testing data that can differ in both test lengths and sample sizes. On the other hand, for *k* = 100 or *k* = 200, the relative speedup was the highest at *n* = 2000 and had a slight drop when *n* increased to 5000 and/or 10000 (see Figures 5 and 6). This may be due to the reason that when *k* gets large (*k* > 50), having an increase of 3000 or 5000 in *n* would result in a significant amount of computation, and therefore CUDA uses proportionally more time to complete the Gibbs sampler.

**Figure 8:**
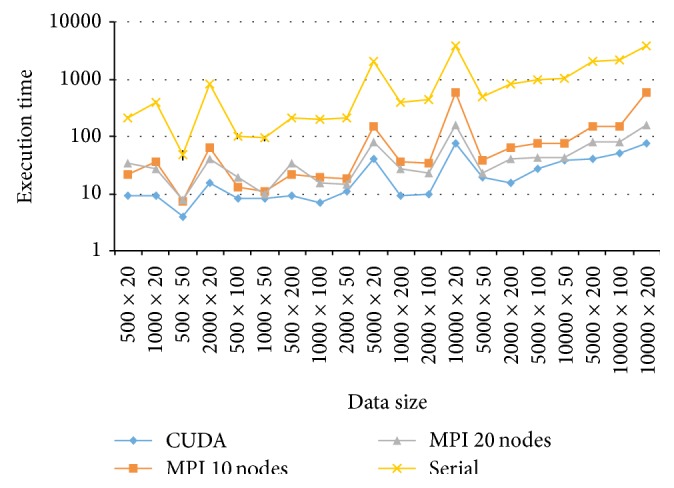
Execution time for implementing MPI and CUDA parallel algorithms of Gibbs sampling for data sizes of *n* × *k*.

To further compare the four implementation methods for all the experimented conditions, the execution times are ordered by the data size and plotted in Figure 8. It is apparent that both GPU- and CPU-based approaches achieve a speedup over the serial implementation, with a clear advantage to the GPU approach. Furthermore, for data of the same size, *n* seems to be the key factor that determines the execution time for each individual implementation. Specifically, a larger *n* tends to result in a longer time in carrying out the Gibbs sampler regardless of the serial or parallel algorithm.

Finally, a comparison of the two parallel implementations in MPI (namely, MPI with 10 nodes and MPI with 20 nodes) suggests that when the data size and especially the sample size *n* are small, the use of fewer number of processing nodes (e.g., 10) is preferred, but when *n* increases, more processing nodes are necessary to achieve an improved speedup. This agrees with the findings of [33].

## 4. Real Data Example

A set of the* College Basic Academic Subjects Examination* [43,* CBASE*] data was used to further illustrate the proposed GPU parallel algorithm. The* CBASE* is a criterion-referenced achievement examination adopted by over 140 colleges and universities across the USA to evaluate knowledge and skills in four subject areas of sophomore-level students (usually after they complete the core curriculum). The data used in this study were from college students who took the LP form of the* CBASE* in years 2001 and 2002. After removing missing responses, there were 3,356 examinees left. The overall* CBASE* exam contains 180 multiple-choice items, with 41 for English, 56 for mathematics, 41 for science, and 42 for social studies. We can assume that all items are measuring the same unified latent trait-academic knowledge and fit the 2PNO IRT model using the proposed algorithm.

The program for implementing this algorithm was developed on a Linux operating system with a NVIDIA K20 GPU. The latter is required to handle desired sample sizes. The program runs from the command line and is available upon request to the first author. For the* CBASE* data, it took the program 40 seconds (less than a minute) to implement the Gibbs sampler with 10,000 iterations. After the burn-in stage of 5,000 iterations, the posterior estimates and Monte Carlo standard errors for the item parameters were obtained and Table 1 displays those for the first 10 items. It is clear from the table that all the Monte Carlo standard errors are small, suggesting accurate posterior estimates. These estimates were also compared with those obtained from the serial implementations in C and via the MATLAB function developed by Sheng [44] and found to be close. It is noted that the latter two implementations took a much longer time, with 17 minutes for the serial C implementation and 33 minutes for the MATLAB function. Again, with a speedup of up to 49×, the GPU approach is practically more attractive and therefore much preferred.

**Figure 9:**
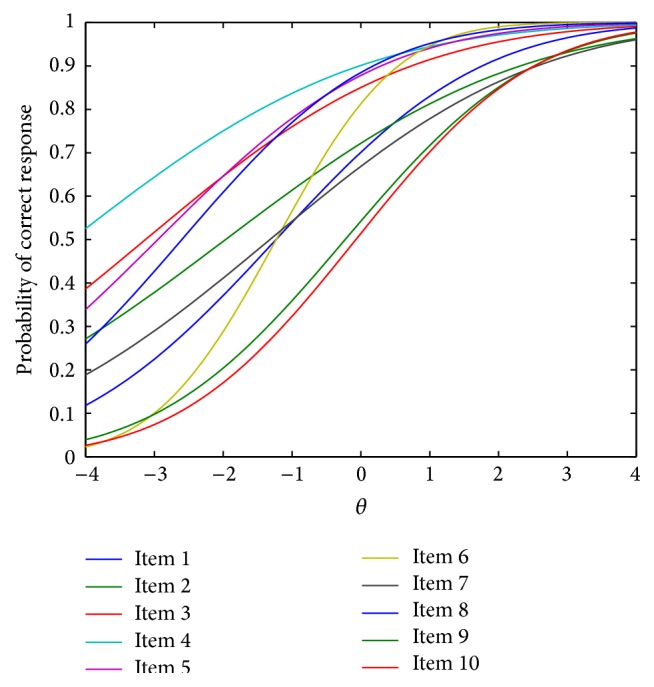
Item response curves for the first 10 items in the* CBASE* data.

**Figure 10:**
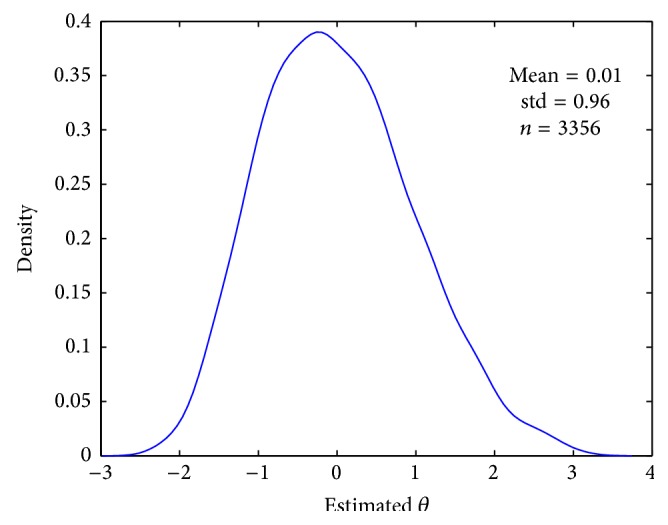
Probability density of the posterior estimates of *n* = 3356 person traits with the* CBASE* data.

**Figure 11:**
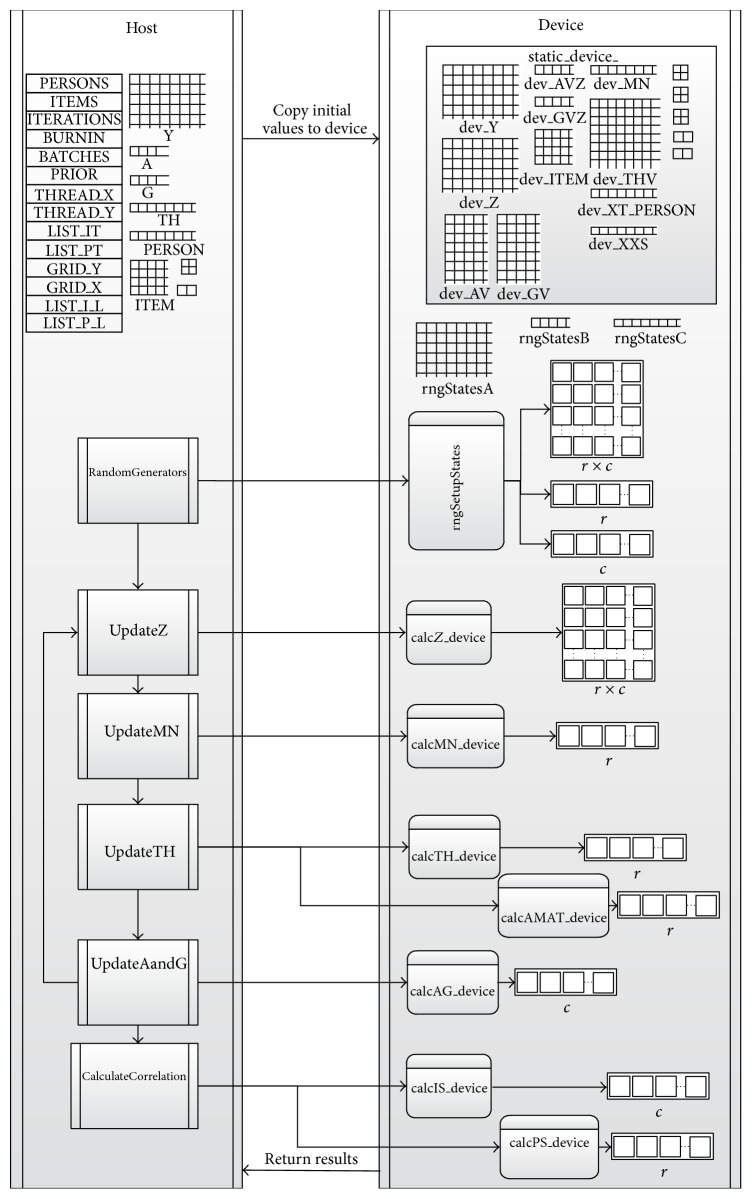
The control diagram between host and device for the developed CUDA program.

Using the estimated item parameters from Table 1 and (2), we can plot the item response curves as shown in Figure 9. A close examination indicates that among the first ten items, item 6 has the largest $\hat{\alpha }$ value (or the steepest curve) and thus is the most discriminating between high and low ability students, whereas item 10 has the largest $\hat{\beta }/\hat{\alpha }$ value (or its curve is on the rightmost) and hence is the most difficult. In addition, the posterior estimates of person parameters for all 3,356 students were obtained and plotted in Figure 10. The density plot has a positive skew, suggesting that in the* CBASE* data, there are more high achieving students ($\hat{\theta }>2$) than low achieving students ($\hat{\theta }<-2$). Also, for those with a medium ability level ($-1<\hat{\theta }<1$), more students are below the mean ($\hat{\theta }=0$) than above it.

## 5. Discussion

This study developed a CUDA GPU-based high performance Gibbs sampling algorithm for the 2PNO IRT model with the purpose of achieving high speedup and efficiency. The algorithm was implemented using the ANSI C programming language and the CUDA interface. The performances were compared with that of the parallel MPI program developed previously. Results indicated that the GPU-based parallel algorithm performed better than the CPU-based algorithm for tests with more than 20 items and that this advantage was more apparent for larger sample size conditions (e.g., *n* > 1000). This further suggests the computational advantage of CUDA-enabled GPU in fitting such IRT models to, for example, large-scale standardized test situations. Moreover, it has to be noted that in the study, the CUDA implementation was realized using one GPU card with 2400 cores, whereas the MPI implementation was realized via the use of a computer cluster. The relatively lower cost of the GPU card makes the proposed approach more cost-effective and convenient for many small research laboratories.

Although this paper only focuses on a specific measurement model, its methodology and results shed light on (1) using GPU to improve efficiency with MCMC algorithms for other models (such as factor analysis models or structural equation models) in general and (2) developing GPU-based Gibbs sampler for more complicated IRT models more specifically. In the IRT literature, the model can be more complex by assuming multiple latent traits, and such tests typically involve more than 20 items. Given this, the GPU-based parallel computing is theoretically more appealing than the CPU-based approach.

In this study, the performance of the GPU program was improved via optimizing global memory accesses and enabling massive thread-level parallelism. Its performance can be further improved by incorporating dynamic parallelism, a nested thread-level parallelism that is available in the CUDA 5 package. CUDA 5 allows the parent kernel to invoke child kernels so that a kernel can dynamically decide the dimension of the to-be-called kernel to achieve an adaptive thread utilization and a better performance [45]. In addition, a kernel can be divided to have multiple streaming data transfer from host to device and to perform computation and data transfer from device to host. This enables (1) the bidirectional data transfer between host and device and (2) kernel computations to be executed concurrently. These strategies together with other optimization techniques such as shared memory, parallel reduction [46], and Open Multi-Processing (OpenMP) interoperability with CUDA [47] can be employed to potentially reduce the total run time.

Finally, this study achieved parallelization of the Gibbs sampler for the 2PNO IRT model through a massive-core GPU computing and compared its performance with the MPI approach developed by [33]. It will also be interesting to consider a different decomposition scheme with MPI such as the 2D decomposition suggested by Georganas [48] or use a hybrid CUDA, MPI, and/or OpenMP parallel programming as recommended by Karunadasa et al. [49–51].

## Figures and Tables

**Table 1:** Posterior estimates and Monte Carlo standard errors (MCSEs) for the first 10 items in the *CBASE* data.

Item	$\hat{\alpha }$	MCSE	$\hat{\beta }$	MCSE	$\hat{\beta }/\hat{\alpha }$
1	0.4280	0.0013	−0.5273	0.0009	−1.2320
2	0.2993	0.0005	−0.5884	0.0004	−1.9659
3	0.3316	0.0002	−1.0379	0.0003	−3.1300
4	0.3061	0.0015	−1.2878	0.0008	−4.2071
5	0.3952	0.0009	−1.1658	0.0014	−2.9499
6	0.7231	0.0018	−0.8883	0.0013	−1.2285
7	0.3302	0.0006	−0.4362	0.0006	−1.3210
8	0.4612	0.0009	−1.1996	0.0008	−2.6010
9	0.4669	0.0007	−0.1065	0.0005	−0.2281
10	0.4943	0.0008	−0.0358	0.0008	−0.0724
